# Supplementary material for: Elevated CO2 alters photosynthesis, growth and susceptibility to powdery mildew of oak seedlings
Source: Biochem J. 2023 Sep 6;480(17):1429–43. doi: 10.1042/BCJ20230002 (PMC10586781; doi:10.1042/BCJ20230002)

Supplem**entary Figure S1**. Visual effects of enhanced CO2 on A) growth at early timepoints and B) powdery mildew resistance in oak represented

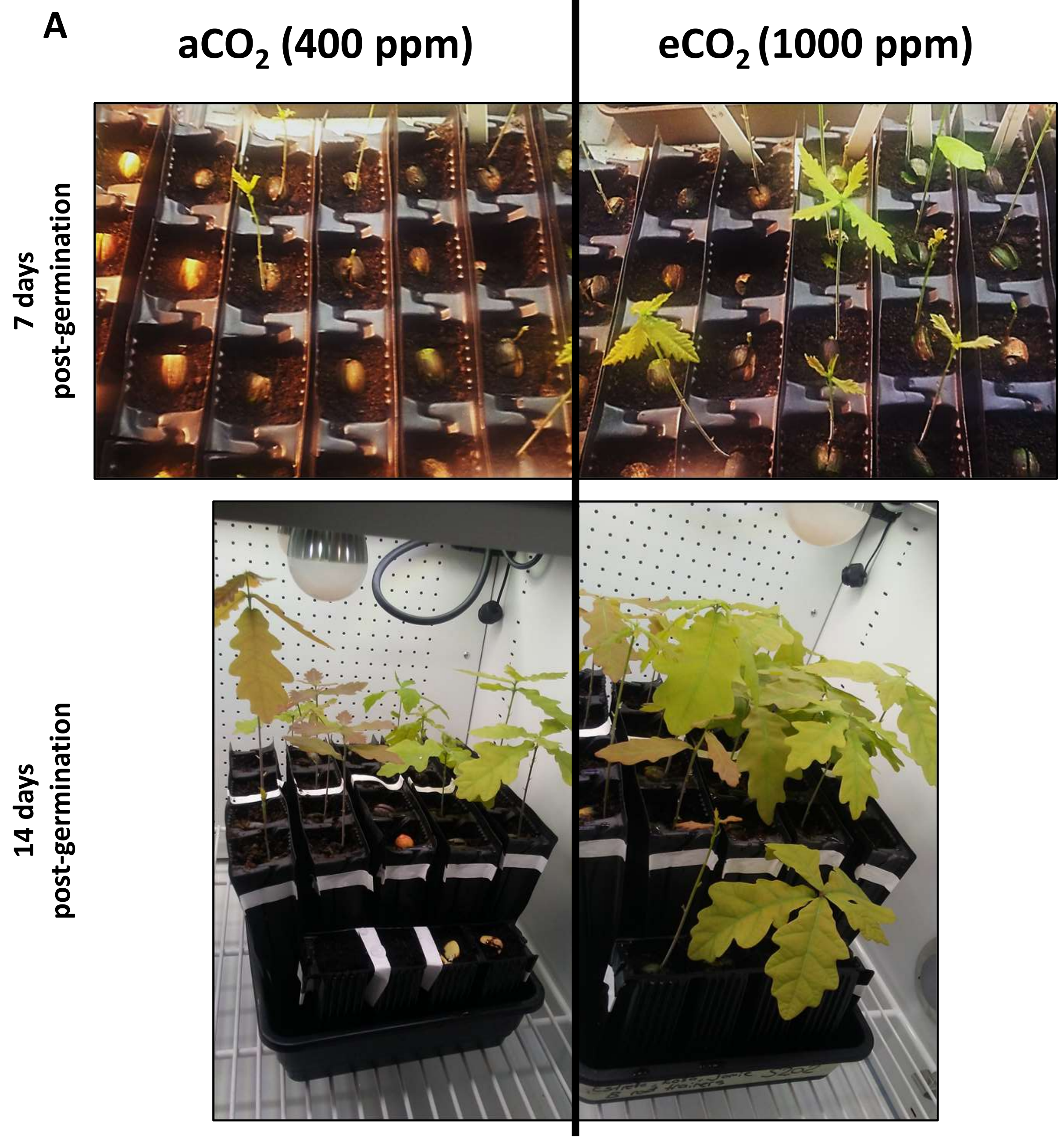

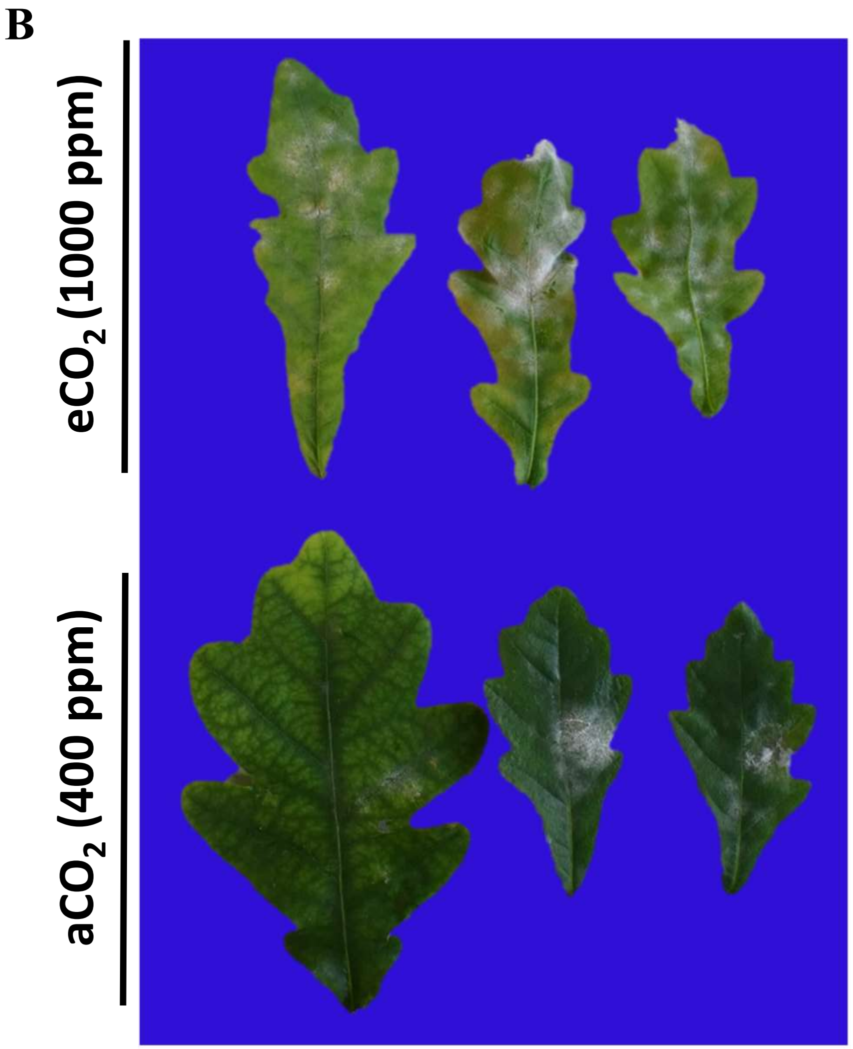

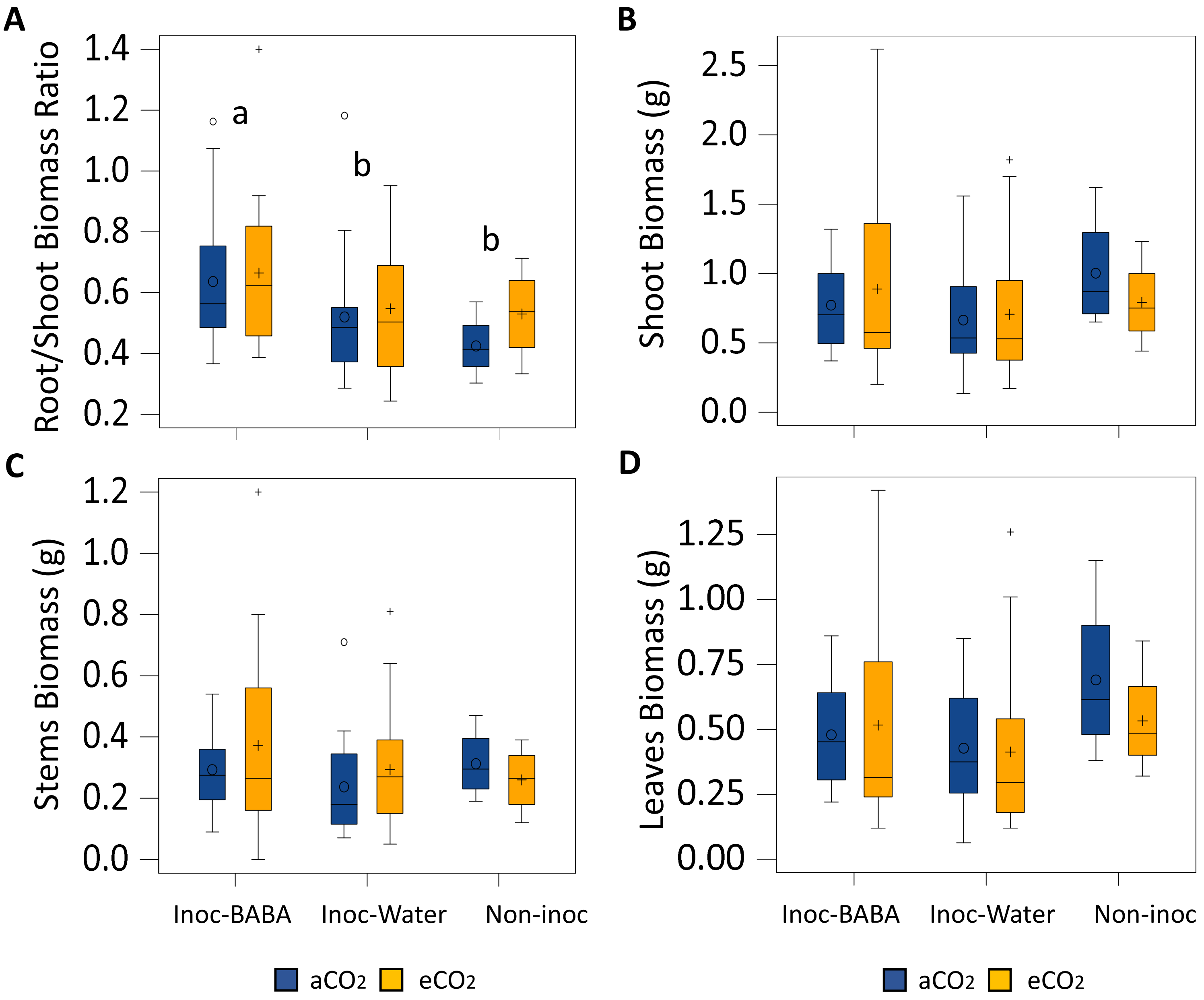

Supplementary Figure S2. Combined effect of enhanced CO2 levels and BABA treatment on biomass allocation in roots/shoot ratio **(A)**, shoots **(B)**, stems **(C)** and leaves **(D)** at the end of the experiment. Lowercase letters inside the figures represent statistically significant differences between groups: Inoc-BABA representing infected seedlings treated with BABA; Inoc-water representing infected seedlings and Non-inoc representing non-infected seedlings (Tukey post-hoc test; $p<0.05$; n=8 for inoculated plants/n=4 for non-inoculated plants).

Supplementary Figure S3. Combined effect of enhanced CO2 and BABA treatment on oak seedlings phenotypes. Inoc-BABA represents infected seedlings treated with BABA; Inoc-Water represents infected seedlings treated with water and Non-inoc represents non-infected seedlings.

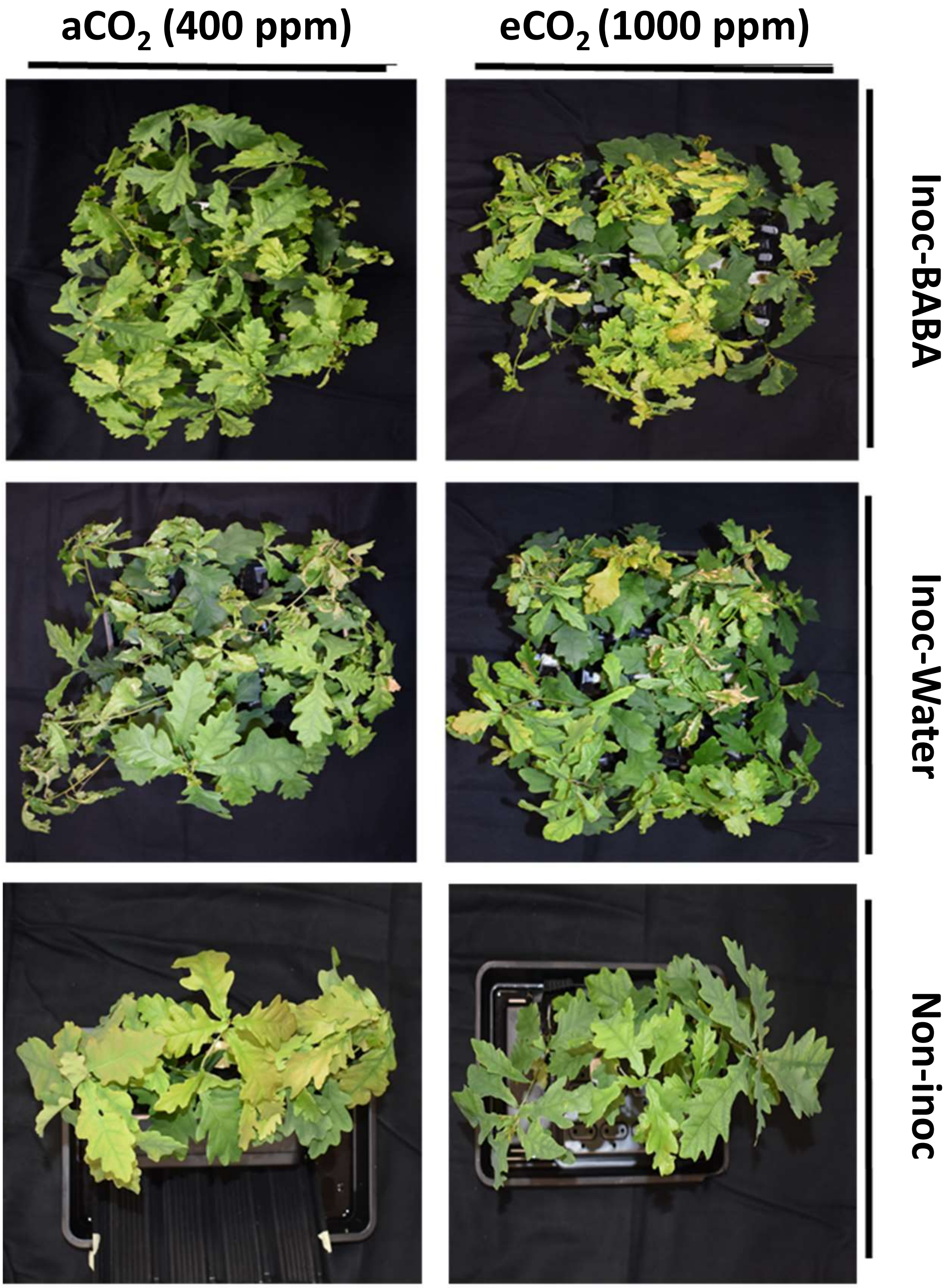

Supplement: Supplementary Material [file BCJ-480-1429-s1.pdf]
